# Exploring the Potential of Glutathione Reductase Overexpression to Improve Tellurium Nanoparticle Production in *Escherichia coli*

**DOI:** 10.3390/ijms26041549

**Published:** 2025-02-12

**Authors:** Jefferson O. Romero, Víctor Castro-Fernández, Estefanía Pérez-Agudelo, Fabián Ávila-Salas, Francisco Mura, Felipe Arenas Salinas

**Affiliations:** 1 Departamento de Biología, Facultad de Química y Biología, Universidad de Santiago de Chile, Santiago 9170022, Chile; jefferson.romero@usach.cl; 2 Departamento de Biología, Facultad de Ciencias, Universidad de Chile, Santiago 7800003, Chile; vcasfe@uchile.cl; 3 Centro de Nanotecnología Aplicada, Facultad de Ciencias, Ingeniería y Tecnología, Universidad Mayor, Santiago 8580745, Chile; tefanypa@hotmail.com; 4 Escuela de Agronomía, Facultad de Ciencias, Ingeniería y Tecnología, Universidad Mayor, Santiago 8580745, Chile; 5 Departamento de Ciencias del Ambiente, Facultad de Química y Biología, Universidad de Santiago de Chile, Santiago 9170022, Chile; francisco.mura@usach.cl

**Keywords:** tellurium nanoparticles (TeNPs), tellurite reductase activity, glutathione reductase, biosynthesis TeNPs, enzyme overexpression

## Abstract

Tellurium nanoparticles (TeNPs) are emerging as valuable materials in various technological and biomedical applications due to their unique physicochemical properties. In general, TeNPs are prepared using chemical methods based on a redox reaction in which strong reducing agents are employed which are often toxic and harmful to the environment. Biological biosynthesis provides a green strategy for substituting the commonly used reducing chemical agents with microorganisms or enzymes. Among the enzymes noted as key players in microbial tellurite reduction, glutathione reductase (GR) has been identified; however, its specific role in enhancing TeNP biosynthesis has yet to be fully elucidated. In this study, we aimed to evaluate the impact of GR overexpression on TeNP production in *Escherichia coli* (*E. coli*). For this purpose, four GR enzymes from different microorganisms identified as tellurite resistant were heterogeneously expressed and purified from *E. coli*. The kinetic parameters for NADPH and oxidized glutathione (GSSG), the native substrates of GR, were determined to evaluate their TR activity under saturated NADPH concentrations. The GR from *Pseudomonas lini* strain BNF22 presented the highest catalytic efficiency for NADPH and exhibited greater TR activity. This enzyme was overexpressed in *E. coli* MG1655 (DE3) and cells overexpressing GR increased the yield of TeNPs in those cells, presenting an increased elemental cell tellurium composition. Our results provide valuable insights for the development of engineered *E. coli* as a platform for TeNP biosynthesis. Using microorganisms as a green strategy for TeNP production, the results of this study highlight the enzymatic mechanisms underlying the role of GR in the biosynthesis of TeNPs.

## 1. Introduction

Tellurium (Te; *Z* = 52) is a metalloid element in group XVI of the periodic table that occurs naturally as a by-product of the processing of copper, gold, lead, and iron, primarily derived from mining [1]. It is classified as a critical element due to the potential risk to the security of its supply. Its use has increased due to the element’s photoconductivity and thermoconductivity, resulting in its application in emerging technologies, such as photovoltaic cells for solar energy conversion and thin-film technologies [2,3,4]. Therefore, due to the natural scarcity of Te and its increasing demand, research efforts have focused on exploring new approaches to the extraction and recycling of Te. Chemical/electrochemical processes and biological reduction have been studied as viable strategies to enhancing tellurium sources [5]. Biological reduction is currently considered an eco-friendly alternative to the traditional chemical processes applied in the metallurgical industry as the costs involved in this process are low and it is easier to perform; reactions can occur under inexpensive conditions using standard culture media at a neutral pH, near-ambient temperature, and atmospheric pressure without sophisticated infrastructure being required. The aim of this process is to improve the crystallinity, morphology, and yield of the quantities obtained after the purification steps required to recover tellurium from biomass [6].

Bacteria and fungi bioreduce the toxic oxyanions of tellurium, tellurite (${TeO}_{3}^{2-}$), and tellurate (${TeO}_{4}^{2-}$) to zerovalent non-pollutant Te^0^, forming tellurium nanoparticles (TeNPs) that accumulate in their cytoplasm or periplasm [7]. This process is a detoxification strategy employed by microorganisms, the mechanism of which has yet to be fully elucidated [8,9]. However, it has been recognized that different bacterial enzymes are directly involved in the reduction process of tellurium oxyanions; for example, glutathione reductase (GR) [10], nitrate/nitrite reductases, dihydrolipoyl dehydrogenase (DLD) [11], flavorubredoxin reductase (NorW), alkyl hydroperoxide reductase (AhpF), thioredoxin reductase (TrxB) [12], mycothione reductase (Mtr) [13], and flagellin (FlaA) [14], among others. In addition, thiopurine methyltransferase (TPMT) [15], superoxide dismutase (SOD), catalase (CAT) [16], alcohol dehydrogenase (YqhD) [17], 6-phosphogluconate dehydrogenase (Gnd) [18], cation/acetate symporter (ActP) [19], and inorganic phosphate transporters (PitA/PitB) [20] participate in the tellurite detoxification process in bacteria through the activation of several mechanisms that help bacteria cells survive when exposed to toxic concentrations of tellurite/tellurate.

In the fields of biotechnology and microbiology, research on the biological reduction of tellurium currently focuses on three main aspects: (1) searching for different microorganisms capable of reducing tellurite and tellurate by forming TeNPs [1]; (2) evaluating the effects of different microorganism culture conditions on TeNP formation [7,21,22]; and (3) engineering bacteria to more efficiently bio convert tellurium into TeNPs by overexpressing proteins known to participate in microbial tellurium detoxification. [13,23,24].

Although GR has demonstrated the highest tellurite reductase (TR) activity among the enzymes that display this activity [12], recently genetic studies deleting the *gorA* gene in *E. coli* strain BW25113 showed that TR activity interconnected the pathways of sulfur metabolism and oxidative stress response, with GR playing a basic role [25]. The effect of overexpressing this enzyme in *E. coli* cultures has yet to be investigated to evaluate the impact on TeNP production by *E. coli*. With this aim in mind, we first characterized and compared four orthologs of GR from microorganisms that have been reported to possess tellurite resistance: BNF08 (*Psychrobacter immobilis*), BNF22 (*Pseudomonas lini*), *E. coli*, and MF01 (*Enterobacter cloacae*) [26,27]. The enzyme with the highest TR activity was selected and overexpressed in *E. coli* and its ability to reduce tellurite was characterized using chemical analyses and microscopy techniques.

The results of this study provide insights into the enzymatic mechanisms that govern the bioreduction of tellurite by GR and demonstrate that overexpression of this enzyme can serve as an effective strategy for engineering strains for use in TeNP biosynthesis.

## 2. Results and Discussion

### 2.1. BNF08 GR Sequence Analysis

We have previously studied the role of GR enzymes in the biosynthesis of TeNPs [12,26,27]. The GR enzymes of the microorganisms *Pseudomonas lini* (BNF22), *Enterobacter cloacae* (MF01), and *E. coli* were cloned, purified, and preliminarily characterized. In this study, we compared these enzymes with an additional GR enzyme, cloned in this study from the BNF08 strain (*Psychrobacter immobilis*), a microorganism that also displays resistance to tellurite [24].

**Figure 1:**
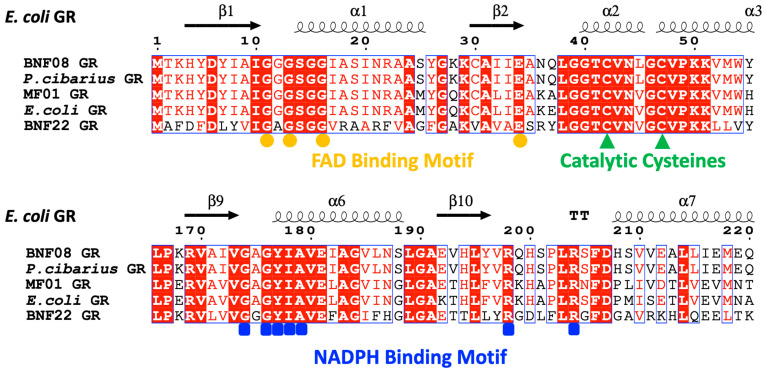
Alignment of the amino acid sequence of BNF08 GR with other GR enzymes from various organisms. The yellow circles indicate the conserved motif for FAD binding, the blue squares mark the NADPH binding motif, and the green triangles highlight the catalytic redox-active cysteine residues.

The sequence analysis of BNF08 revealed a protein consisting of 451 amino acids. Alignment of the amino acid sequence with the other GR enzymes, performed using the MultiSeq tool [28], indicated sequence identities of 67.8% with MF01 GR, 69.7% with *E. coli* GR, and 41.9% with BNF22 GR. The alignment also revealed that BNF08 GR conserved the two active site electron acceptors, FAD-binding motif GxGxxG(x17)E and the redox-active disulfide in addition to the NADPH-binding motif (GXGYIAX18RX5R) (Figure 1); all of these motifs are characteristic of flavoenzymes and they are present in other GR orthologs that also conserve the cysteine residues described as crucial in TR activity [12,29]. Initial studies from our laboratory, based on 16S rRNA sequencing, identified strain BNF08 as being related to *Psychrobacter immobilis* [27]. Contrasting GR-BNF08 with other GR sequences using BLASTp revealed a 99.1% identity with GR sequences reported for *Psychrobacter cibarius* (WP_201502771.1).

### 2.2. Spectral and Molecular Weight Evaluation for Purified GR Enzymes

**Figure 2:**
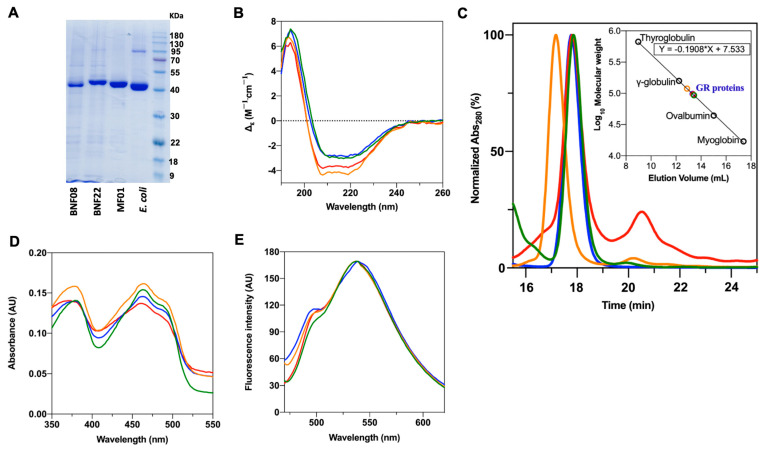
Spectral and molecular weight evaluation for purified GR enzymes: 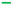 *E. coli* GR, 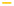 BNF22 GR, 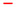 BNF08 GR, and 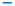 MF01 GR. (**A**) SDS-PAGE gel. (**B**) CD spectra. (**C**) Results of the size exclusion chromatography. The molecular weight of GR enzymes was determined to be ≈110 kDa. (**D**) Absorption spectra under reduced conditions. (**E**) Fluorescence emission spectra.

All of the proteins (GR from BNF08, BNF22, MF01, and *E. coli*) were recombinantly expressed in *E. coli*, purified, and characterized. The elution peak for all proteins migrated as a single band in sodium dodecyl sulfate polyacrylamide gel (SDS-PAGE) with a molecular weight close to 50 kDa (Figure 2A); they presented a yellow color from the flavin prosthetic group covalently bound to the enzyme. Far-ultraviolet circular dichroism (CD) spectra revealed that all GRs exhibited the typical spectra profiles characteristic of α/β proteins, with a high α-helix, consistent with previous reports on GR [30] (Figure 2B). Size exclusion chromatography using a Superdex^TM^ 200 column (GE Healthcare Life Sciences, Piscataway, NJ, USA) revealed that all of the proteins are homodimers of approximately ≈110 kDa (Figure 2C). The absorption spectrum is characteristic of flavoprotein, displaying peaks at ≈378 nm and ≈460 nm (Figure 2D) [31]. The emission spectra demonstrated the presence of flavin adenine dinucleotide in a reduced form (FADH_2_) bound to all of the enzymes (maximum peaks close to 495 nm and 540 nm) [31,32] (Figure 2E).

### 2.3. GR Kinetic Studies and TR Activity

To compare the native activity of GR and TR, we performed a kinetic characterization. The kinetic parameters of the purified GR enzymes are provided in Table 1. The optimal pH to evaluate GR activity was 7.0 for all of the proteins. The evaluations were conducted at the optimal growth temperatures reported for each microorganism: 25 °C for BNF08 and BNF22 and 37 °C for *E. coli* and MF01. The catalytic efficiency (*K*_cat_/K_M_) for NADPH of GR from BNF22 was twice that of the other enzymes studied; in comparison, for its native substrate, oxidized glutathione (GSSG), it presented a difference of an order of magnitude. The K_M_ values for *E. coli* GR presented similar values to those previously reported [33].

**Figure 3:**
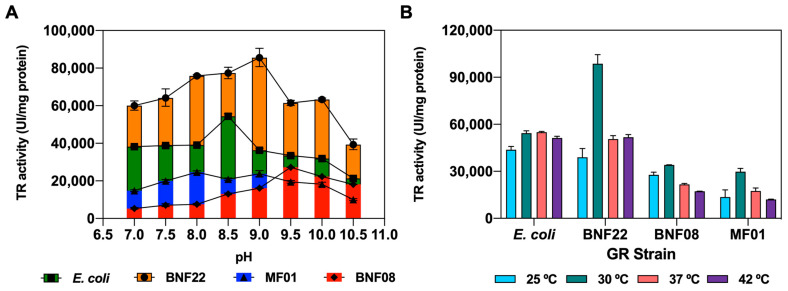
Effect of (**A**) pH and (**B**) temperature on the TR activity of the purified enzymes.

The characterization of the GR kinetic parameters provided the saturated concentration value of NADPH required to evaluate TR activity (15 times K_M_). Then, the optimal pH values for TR activity were elucidated: 8.5 for *E. coli*, 9.0 for MF01 and BNF22 GR, and 9.5 for BNF08 GR (Figure 3A). The importance of the motif CXXC in TR activity has been described and the presence of a thiol group (not a strong nucleophile) defined the reactivity of GR to tellurite at the basic pH values [12,34]. A neutral to basic pH has also been described as the optimal pH condition in other proteins presenting TR activity [13,14,24]. The optimal temperature for TR activity was 30 °C for all of the proteins evaluated (Figure 3B).

The GR from the BNF22 strain was the enzyme with the highest catalytic efficiency to reduce NADPH. The *k*_cat_/K_M_ ratio for BNF22 GR was 8-, 11-, and 12-fold higher that of MF01, *E. coli*, and BNF08 GR, respectively. Additionally, it presented the highest TR activity at 2 times higher than *E. coli* GR and 3 times higher than MF01 and BNF08 GR. 

The authors of a previous study comparing the subunit E3, dihydrolipoyl dehydrogenase (DLD) of the pyruvate dehydrogenase (PDH) complex from different tellurite-resistant microorganisms established that DLD from highly resistant microorganisms displayed elevated TR activity [11]. In this study, no such correlation was observed. In contrast, we found that GR enzymes with high reductase activity also demonstrated elevated TR activity. Based on the proposed mechanism for GR described by Berkholz et al. [35], we propose that GR enzymes that are more efficient in reducing NADPH will more effectively activate catalytic cysteines through flavin reduction, thereby enhancing tellurite reductase (TR) activity.

It is hypothesized that higher TR activity may arise from shorter distances between the catalytic atoms within the active site, facilitating more efficient interactions during the catalytic process [12]. In this study, we analyzed the distance between the hydrogen atom of FADN5 and the sulfur atom of Cys47 during 50 ns of molecular dynamics (MD) simulations of model structures of GR enzymes studied generated with Alphafold 3 web server Beta [36]. The MD simulations were performed with the DESMOND program [37,38] and the force field OPLS_2005 was selected. Among the GR enzymes studied, the GR from BNF22 exhibited the shortest distance between these catalytic atoms compared to the other GR enzymes analyzed in this study (see Supplementary Material Information Table S2, Figure S1). Our experiment allowed us to elucidate a potential atomic interaction that may account for the higher reductive capacity observed in GR from BNF22.

### 2.4. In Vivo TeNP Synthesis in E. coli and Characterization Results

The results of previous studies have shown that the overexpression of TPMT and TrxR in *E. coli* increases bacterial tolerance to tellurite and enhances tellurite reduction [15,23,24]. Based on these findings, we evaluated the effect of the overexpression of BNF22 GR, the enzyme with the highest TR activity among those studied. We selected *E. coli* MG1655 (DE3) as the host strain due to its tellurite sensitivity, which facilitated the monitoring of changes in the color of the cultures during growth; additionally, this strain exhibits a higher tolerance to growth under stressed conditions compared to other *E. coli* strains [39].

**Figure 4:**
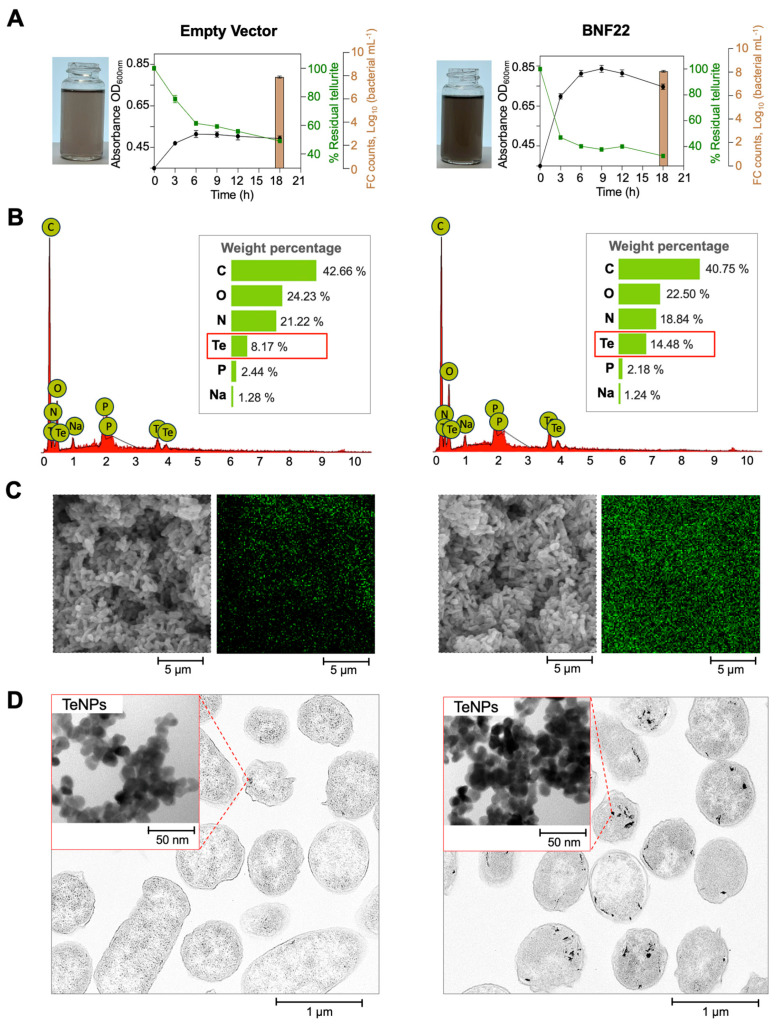
Characterization of the in vivo reduction of tellurite in *E. coli* MG1655 (DE3) overexpressing BNF22 GR (**right**) or with an empty vector (**left**). (**A**) Growth (black), residual tellurite (green), and flow cytometry FCM cell count at 18 h (brown). (**B**) Energy-Dispersive X-Ray (EDX) spectra for cells harboring the empty vector (left) and BNF22 GR (right) showing cell tellurium content (red box). (**C**) Scanning Electron Microscopy microphotographs for cells harboring the empty vector and BNF22 GR and corresponding images of the EDX elemental mapping results showing the tellurium localization (green color). (**D**) Transmission electron microphotographs of ultrathin cell sections of *E. coli* overexpressing the empty vector or BNF22 GR. The red boxes in the TEM images provide higher magnification views highlighting the TeNPs aggregates formed inside the cells.

*E. coli* MG1655 (DE3) overexpressing BNF22 GR did not present an increase in its resistance to tellurite. We evaluated the minimum inhibitory concentration (MIC) on M9 media in the concentration range from 0 to 2.5 mM K_2_TeO_3_, and the strains harboring an empty vector or BNF22 GR showed the same MIC value (1.25 mM). The only difference observed in our experiments was that the strain overexpressing BNF22 GR exhibited s darker coloration in the cultures compared to the control strain (Figure 4A).

The cultures overexpressing BNF22 GR (verified through SDS-PAGE) presented a higher OD_600_ value for those harboring an empty vector (Figure 4A). The high OD_600_ value could be related to the major biomass present in the cultures; however, the number of cells counted via flow cytometry (FCM) was demonstrated to be similar in both cultures at 18 h (Figure 4A). Thus, the major OD_600_ nm registered in the stationary phase of cell growth is likely due to interference from the scattering and absorption of elemental tellurium as the maximum absorption of elemental tellurium (520 nm) is close to 600 nm. Furthermore, a higher percentage of dead cells was found in cultures overexpressing the GR of BNF22; the presence of TeNPs has been shown to affect the viability of *E. coli* cells by impacting membrane integrity and/or increasing the proportion of reactive oxygen species in the cell, causing oxidative damage to biological macromolecules [40]. The residual tellurite in the medium presented a significant difference between the studied strains. The strain overexpressing BNF22 GR reduced 20% more tellurite than the strain harboring the empty vector (Figure 4A).

The scanning electron microscopy (SEM) coupled to EDX analysis results revealed the difference in the cell tellurium content, accounting for a weight percentage of tellurium of ~14.8% for the strain overexpressing BNF22 GR and 8.17% for the control strain (Figure 4B). The elemental mapping results corroborated the abundance of elemental tellurium in the cell samples, showing a uniform distribution and a higher intensity of the strain overexpressing BNF22 compared to the control strain (Figure 4C). SEM microphotographs of each sample showed the absence of extracellular material (Figure 4C), as has been reported for other microorganisms that are involved in tellurite bioreduction [23,26], suggesting that elemental tellurium is present inside the cells and likely forms the deposited TeNPs.

The TEM analysis results obtained in this research verified the presence of electron-dense nanoparticles inside the cells (Figure 4D). These structures were likely TeNPs and were localized in cytoplasmic and periplasmic regions, as previously reported in *E. coli* [7]. A low quantity of nanostructures was observed in the control strain in ultrathin cell sections; in addition, a high TeNP content was observed for the strain overexpressing BNF22 GR (Figure 4D).

The above findings are consistent with prior studies on microbial nanoparticle synthesis, in which GR activity in *Pseudomonas stutzeri* TS44 was crucial for the biosynthesis of selenium nanoparticles and the formation of CdSe quantum dots [41]. In contrast with previous studies linking microbial resistance levels to tellurite and the TR activity of proteins involved in its reduction [11,23,24], our results demonstrate that the overexpression of GR from the BNF22 strain contributes to TeNP production but does not influence the resistance of *E. coli* to tellurite.

### 2.5. Nanostructure Purification and Characterization

**Figure 5:**
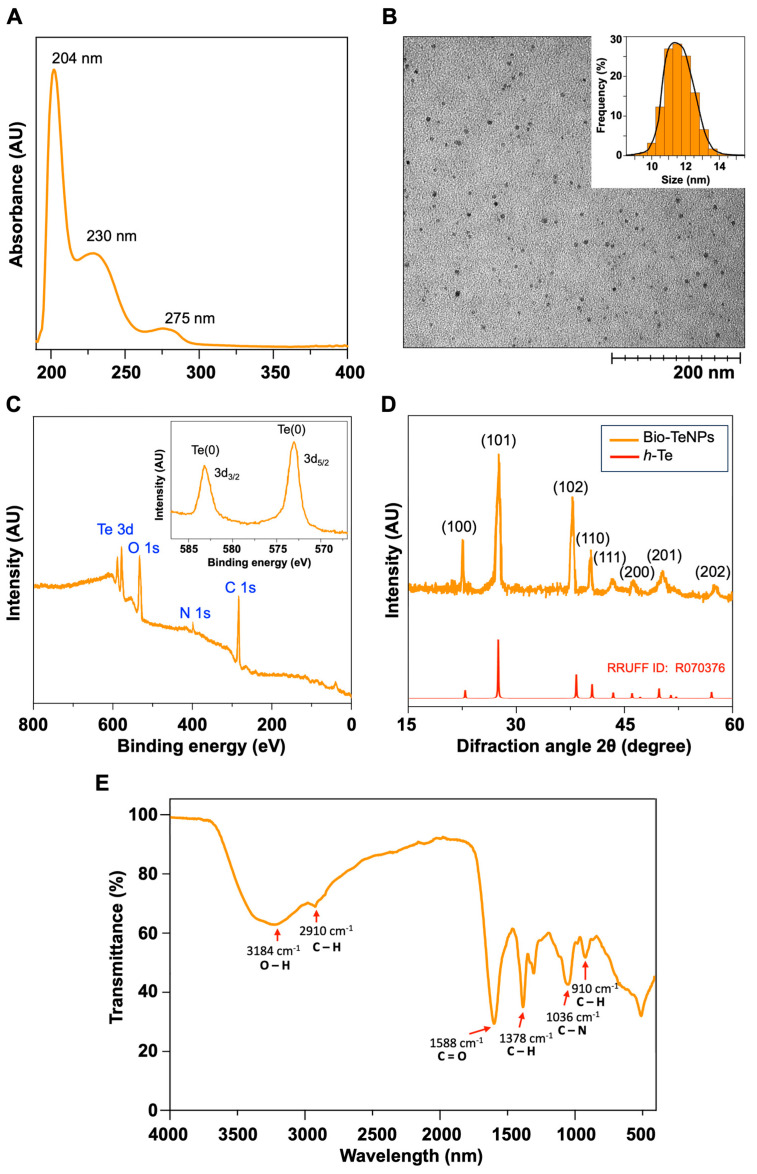
Characterization of TeNPs purified from *E. coli* cultures overexpressing GR from BNF22. (**A**) UV–visible spectrum of TeNPs. (**B**) Transmission electron microphotograph of purified TeNPs. Insert picture shows average size distribution for TeNPs from TEM microphotograph. (**C**) XPS spectra of TeNPs produced. Insert picture shows Te 3d core-level spectrum of TeNPs. (**D**) XRD pattern for biosynthesized TeNPs in orange color and reported XRD pattern of hexagonal tellurium phase (*h*-Te) in red color (RRUFF ID: R070376). (**E**) FTIR spectra of TeNPs generated from *E. coli* cultures.

The TeNPs produced by the strain overexpressing GR form BNF22 were characterized. After ultracentrifugation different fractions were recollected, all of them presented the characteristic UV–visible spectrum (Figure 5A): one band observed in the spectrum corresponding to plasmon resonance occurred at 204 nm, indicating the formation of TeNPs [42], another band at 230 nm denoted protein conjugation with TeNPs, and there was an absorption peak at 275 nm, which was due to the valence band (π-bonding triplet) to the conduction band (π-antibonding triplet) [43].

Transmission electron micrographs (TEMs) revealed that TeNPs have an average size of approximately 12 nm (Figure 5B). In contrast, dynamic light scattering (DLS) showed an average size of about 30 nm (Figure S2A), a discrepancy primarily attributed to the differing principles of these techniques. While TEMs provide detailed structural information on the inorganic core, DLS measures the hydrodynamic diameter in solution, including any biomolecular or organic coatings on the nanoparticle surface. Consequently, the larger size observed by DLS indicates the presence of biological residues coating the TeNPs. Additionally, aggregates larger than 100 nm were detected (Figure S2B), likely due to sample treatment during purification. Exposure to organic solvents, elevated temperatures, or suboptimal dissolution conditions can cause partial degradation and aggregation, ultimately affecting the purity, complexity, and size distribution of the final sample. These findings underscore a common challenge in biosynthesizing nanomaterials: the need to optimize purification protocols to ensure the production of stable nanoparticles with homogeneous size and shape distributions [6,44].

X-ray photoelectron spectroscopy (XPS) analysis was carried out to confirm the reduction of K_2_TeO_3_ forming TeNPs in *E. coli* overexpressing GR from BNF22. Figure 5C shows the XPS wide-scan spectra for purified TeNPs. Five peaks at 282.24, 398.01, 532.72, 572.91, and 583.04 eV are observed. The two peaks at 572.91 and 583.04 eV binding energies correspond to Te 3d_5/2_ and Te 3d_3/2_ and the distance between the peaks is approximately 10 eV [45]. These data detailed indicate that the purified TeNPs are formed with elemental tellurium (Te(0)) and organic compounds C, O, and N. Compared to other TeNPs biosynthesized by *Shewanella* sp. strains CNZ-1 and FDL-2, a representative signal for Te (IV) was not detected in XPS spectra, indicating a higher composition of Te (0) in the TeNPs here biosynthesized [9,46].

The crystallographic structure of the generated TeNPs was established from X-ray diffraction (XRD). The XRD pattern of the biosynthesized TeNPs shows diffraction peaks corresponding to the (1 0 0), (1 0 1), (1 0 2), (1 1 0), and (2 0 1) reflections correlated to the hexagonal phase of tellurium, which are consistent with the reported XRD pattern (*h*-Te) ID R070376 from RRUFF database [47], as shown in Figure 5D. The hexagonal phase of Te crystal was also reported in the biosynthesis of TeNPs using *Bacillus* sp. strain BZ [42].

Fourier-transform infrared spectroscopy (FTIR) was performed to analyze the functional organic groups attached to the surface of the TeNPs acting as reducing and capping agents. Figure 5E shows a FTIR spectrum of biogenic TeNPs with some typical absorption bands; the strong absorption at 1588 cm^−1^ and 1036 cm^−1^ corresponded to carbonyl (C=O) and C–N stretching vibrations, respectively. The stretching vibrations of O–H groups could be assigned to the broad band noted at 3176 cm^−1^. The bands at 2910 cm^−1^, 1378 cm^−1^, and 910 cm^−1^ were related to hydrocarbon C–H stretching vibrations. Overall, it was concluded that biosynthesized TeNPs may contain lipids or proteins. Other reports indicated the presence of biological residues in the surface of TeNPs biosynthesized with bacterial cells [42,48,49].

The previous data presented here indicate that the overexpression of GR in *E. coli* under the tested experimental conditions results in the formation of small (~12 nm) crystalline tellurium nanoparticles (TeNPs) inside the cells. These nanoparticles are composed of elemental tellurium, with organic compounds of bacterial origin probably attached to their surface.

## 3. Materials and Methods

### 3.1. Strains and Plasmids

The bacterial strains and plasmids used in this study are listed in Table S1. *E. coli* DH5*α* was used in plasmid construction and propagation and *E. coli* C41 (DE3) was used in protein expression and purification. Assays of GR overexpression were performed in *E. coli* MG1655 (DE3); the strain was purchased from Addgene under the bacterial strain reference number #37854 [50].

The GR gene of strain BNF08 was amplified from chromosomal DNA via the polymerase chain reaction (PCR) using the respective primer pair forward 5′TTTCATATGACAAAA-CATTATGATTATATCG3′ reverse 5′TTTAAGCTTACGACGCATTGTCA3′ and genomic DNA purified using the Wizard^®^ genomic DNA purification kit (Promega, Madison, WI, USA). After digestion of the PCR products with *Nde*I and *Hind*III enzymes, the fragments were cloned into a pET21b (+) vector (Novagen, Madison, WI, USA) treated with the same restriction enzymes, creating the plasmid pET-21b-GR-BNF08. This construct was verified by the sequencing services at the Unidad de Secuenciación y Tecnologias Ómicas, Universidad Católica de Chile, Santiago, Chile.

Lastly, the transformation of the described plasmids was performed with chemocompetent *E. coli* C41 (DE3) cells plated on LB plates containing 30 μg/mL of ampicillin or 35 μg/mL of chloramphenicol, generating the strains detailed in Table S1.

### 3.2. Purification of Recombinant Glutathione Reductase (GR) Enzymes

Recombinant GR proteins were purified electrophoretically from *E. coli* strain C41 (DE3) transformed with each plasmid. A total of 1 L of Terrific Broth (TB) medium containing 100 μg/mL of ampicillin or 35 μg/mL of chloramphenicol was inoculated with 1 mL of overnight cell culture of the transformed *E. coli* strains and then cultured at 37 °C with stirring (200 rpm) until the culture reached an OD_600_ of 0.5 (at 600 nm). The culture was then induced with 1 mM of isopropyl β-D-1-thiogalactopyranoside (IPTG) for 16 h at 37 °C. The cells were harvested by centrifugation at 4 °C (8000× *g*, 15 min). Prior to cell disruption, the pellet was resuspended in 50 mL of binding buffer (sodium phosphate–buffer solution (20 mM, pH 7.4) containing 500 mM of NaCl, 1 mM of β-Mercaptoethanol, and 20 mM of imidazole) and sonicated on ice in a Branson Digital sonifier^®^ (Branson Ultrasonic Corporation—Danbury, CT, USA) using 40% of maximal amplitude during 5 cycles of 3 min (20 s on/30 s off). Thereafter, the suspension was centrifuged at 18,000× *g* for 30 min at 4 °C to obtain the crude extract. The crude extract was applied at a flow rate of 1.5 mL/min to a HisTrap HP column (5 mL) (GE Healthcare, Uppsala, Sweden), pre-equilibrated with binding buffer, and washed with 10 column volumes in a ÄKTA^TM^ prime plus system (GE Healthcare, Uppsala, Sweden). A 20–500 mM imidazole gradient was then passed through the column, 1.5 mL fractions were collected, and the enzymatic activity of GR was immediately measured while being maintained on ice. Fractions with higher activity were pooled and dialyzed for 16 h at 4 °C in sodium phosphate buffer (20 mM, pH 7.4), 200 mM of NaCl, and 1 mM of β-Mercaptoethanol. Lastly, one cycle of centrifugation at 4 °C (8000× *g*, 15 min) was performed to eliminate protein precipitates before storage with 30% glycerol at −20 °C.

### 3.3. Characterization of Recombinant Glutathione Reductase Enzymes

#### 3.3.1. Molecular Weight Determination and Spectral Properties

The molecular weight of each purified GR enzyme was determined via size exclusion chromatography using a Superdex^TM^ 200 (GE Healthcare Life Sciences, Piscataway, NJ, USA). The mobile phase used was sodium phosphate buffer (20 mM, pH 7.4), 200 mM of NaCl, and 1 mM of β-Mercaptoethanol. The elution times were compared to the gel filtration protein standard (#151-1901, Bio-Rad, Mississauga, ON, Canada).

The structural integrity of the purified GR enzymes was evaluated via UV–visible, fluorescence, and circular dichroism (CD). The UV–visible spectrum for each protein were recorded using a photodiode array spectrophotometer (Hewlett Packard/Agilent 8453, Santa Clara, CA, USA) at 20 °C. To evaluate the absorption spectra, 10 μM of freshly purified protein dissolved in 50 mM of sodium phosphate buffer at pH 7.4 was reduced with 1 mM of NADPH. Emission spectra were recorded using a Jasco FP-8300 spectrofluorometer (Jasco Corp., Tokyo, Japan). A total of 4 μM of freshly purified protein dissolved in 50 mM of sodium phosphate buffer at pH 7.4 was excited with polarized light at 450 nm and spectral data were collected from 470 nm to 620 nm. The CD spectra were measured on a JASCO J-1500 spectrophotometer (Jasco Corp., Tokyo, Japan) using a final protein concentration of 0.2 mg/mL in 50 mM of sodium phosphate buffer at pH 7.4. Measurements were performed in a 0.1 cm quartz cuvette, with a scanning speed of 50 nm/min from 190 to 250 nm, and three spectra accumulations were performed for each sample.

#### 3.3.2. GR Kinetic Studies and Tellurite Reductase (TR) Activity

GR activity was evaluated using a Jasco V-650 spectrophotometer (Jasco Corp., Tokyo, Japan) at the optimal growth temperatures reported for each microorganism: 25 °C for BNF08 and BNF22 and 37 °C for *E. coli* and MF01 strains [23,24]. The reaction mix (500 μL) included 420 μL of 100 mM sodium phosphate buffer (pH 7.4), 25 μL of 20 mM GSSG, and 25 μL of 8 mM β-nicotinamide adenine dinucleotide phosphate in reduced form (NADPH). The reaction was initiated by adding 10 μL of 2 mM enzyme. One unit of enzyme activity was defined as the amount of GR that oxidizes 1 μM NADPH per minute. The absorbance variation at 340 nm was followed and an extinction coefficient of 6.22 mM^−1^cm^−1^ for NADPH was used.

Kinetic constants were calculated from the initial rates in assays with 1 mM GSSG or 200 μM NADPH as fixed substrate concentrations. The K_M_ and *k*_cat_ values for NADPH and GSSG were determined from a non-linear Michaelis–Menten adjustment in GraphPad Prism version 8. GR activity was determined at the optimal pH for each enzyme and the different NADPH (20 to 300 μM) or GSSG (1 to 800 μM) concentrations.

Tellurite reductase activity (TR) was measured similarly to previous reports [12]. Briefly, 130 μL of buffer was pre-mixed with NADPH in saturated concentrations (15 times the Km value for each enzyme), 10 μL of 20 mM K_2_TeO_3_, and 20 μL of 10 mM β-Mercaptoethanol. A total of 20 μg of the enzyme was added and the production of elemental tellurium was monitored at 500 nm for 40 min using a Tecan Infinite^®^ M200 PRO plate reader (Tecan, Maennedorf, Switzerland). One enzyme unit was defined as the amount of enzyme required to increase the OD_500_ by 0.001. The effect of pH and temperature on tellurite reduction was also evaluated for each enzyme.

### 3.4. In Vivo TeNPs Synthesis and Characterization

The in vivo synthesis of TeNPs was performed using *E. coli* MG1655 (DE3) harboring a pET101-BNF22-GR vector or the vector-empty pET101/D-TOPO^TM^ (Invitrogen, Carlsbad, CA, USA). These strains grew to the exponential phase (OD_600_ ≈ 0.35) in M9 media at 180 rpm and 37 °C; thereafter, induction and tellurite exposition were conducted with the addition of 1 mM of IPTG and 0.3125 mM of K_2_TeO_3_. Cell growth at OD_600_ was monitored for an additional 18 h. The samples were then withdrawn to evaluate tellurite reduction (as a % of residual tellurite) by adapting a methodology described previously [51]. The number of cells was quantified via flow cytometry (FCM) using a LIVE/DEAD BacLight bacterial viability and counting kit (Invitrogen, Thermo Fisher, Waltham, MA, USA), with the samples analyzed according to the supplier’s specification via a BD FACSCANTO II flow cytometer (Becton Dickinson, Mountain View, CA, USA).

Tellurium composition and elemental mapping were determined via SEM/EDX analysis of the cell samples at 18 h; the cells were dehydrated with mixtures of 30, 50, 70, 90, and 100% ethanol/water. After critical point drying, the samples were gold coated using a Jeol Smart Coater (Jeol Ltd., Peabody, MA, USA) before analysis using a Phenom model ProX Desktop SEM microscope (Phenom World, Eindhoven, The Netherlands) integrated with an EDX detector at the Centro de Nanotecnología Aplicada, Universidad Mayor, Huechuraba, Chile.

TeNPs synthesized in vivo were observed in ultrathin cell sections via TEM using a Philips Tecnai 12 microscope (FEI/Philips Electron Optics, Eindhoven, The Netherlands) to 80 kV at the Unidad de Microscopía Avanzada (UMA), Pontificia Universidad Católica de Chile, Santiago, Chile.

### 3.5. Tellurium Nanoparticles Purification and Characterization

The strain overexpressing BNF22 GR was grown to an OD_600_ ~0.35 in M9 medium, treated with 0.3125 mM K_2_TeO_3,_ and incubated for 18 h at 37 °C and 175 rpm. Cells were harvested by centrifugation at 4000× *g* for 10 min, then suspended in 50 mL of 50 mM Tris-HCl buffer pH 7.4 and sonicated on ice for 5 min. Consecutively, 3 washes were performed with 50 mM Tris-HCl buffer pH 7.4 and 0.5% SDS. The final pellet was suspended in 1 mL 50 mM Tris-HCl buffer pH 7.4, where 800 μL of this solution was deposited on top a 20–60% sucrose gradient column. Centrifugation was performed at 300,000× *g* for 3 h in a Sorvall WX 100+ ultracentrifuge (Thermo Fisher, Waltham, MA, USA) using a swinging bucket rotor model AH-650 (Thermo Fisher, Waltham, MA, USA) and 200 μL fractions were collected. The UV–visible spectra of all fractions were recorded using a photodiode array spectrophotometer (Hewlett Packard/Agilent 8453, San Clara, CA) at 20 °C. For fractions in which the spectral feature of tellurium nanostructures was determined, acetone in relation to 1:1 was added. The organic phase was transferred to glass vials and 2% (*v*/*v*) HNO_3_ was added. Overnight incubation at 50 °C dried the solvent and allowed solid-phase nanostructures to be obtained. For observation of TeNPs by TEM, 1 mM of nanostructure solutions were prepared and drops of these solutions were poured onto carbon-coated copper grids. TEM observations were performed on a Talos F200C G2 instrument at the Unidad de Microscopía Avanzada (UMA), Pontificia Universidad Católica de Chile, Santiago, Chile. Finally, TEM photographs were processed using the ImageJ software, version 1.54g [52]. The hydrodynamic diameter of TeNPs was determined using a Zetasizer Nano ZS Marlvern instrument (Malvern Instrument, Malvern, UK) at 20 °C, Laboratorio de Cinética y Fotoquímica. Facultad de Química y Biología, Universidad de Santiago de Chile.

For FTIR, XPS, and XRD characterization, a biosynthesized TeNP sample was dried under vacuum before analysis. The IR spectra were noted at a wave number range of 400–4000 cm⁻^1^ using a Spectrum Two FTIR spectrometer (Perkin Elmer, Waltham, MA, USA). XPS analysis was performed in a SPECS XPS instrument (Specs Group, Berlin, Germany) with a PHOBIOS analyzer, a 1D-DLD detector, and an Al anode as an X-ray source (1486.71 eV). Wide scan spectra were recorded using an energy step of 0.5 eV and a pass energy of 40 eV, while the specific core-level spectrum (Te 3d) was recorded using an energy step of 0.1 eV and a pass energy of 20 eV. XRD was carried out with a RIGAKU Multipurpose SmartLab model diffractometer (Rigaku Corporation, Tokyo, Japan) with a Theta–Theta Bragg–Brentano geometry goniometer and coupled to a solid-state detector D/teX Ultra 250 for data acquisition. The coherent X-ray beam was generated using a copper tube target (Cu Kα λ = 1.5418 Å) at 40 kV/30 mA. The measurement was achieved between 15–60° (2θ), with a step of 0.02° and a scanning speed of 1°∙min^−1^.

## 4. Conclusions

The results of this study demonstrate the potential of GR overexpression to enhance the production of TeNPs in *Escherichia coli*. The results highlight the relationship between GR activity and TeNP biosynthesis, emphasizing that GR enzymes with high catalytic efficiency for reducing NADPH also exhibit elevated TR activity, resulting in the formation of smaller spherical TeNPs.

These results highlight the role of GR in TeNP biosynthesis and suggest that optimizing this process in microbial systems could enable sustainable and scalable nanoparticle production, providing an eco-friendly alternative to traditional methods.

Despite these promising outcomes, further research is needed to address key limitations. Future work should explore alternative microbial hosts with a higher resistance to tellurite, coupling GR in TeNP bio-catalytic routes, optimizing large-scale bioproduction processes, and refining purification protocols of TeNPs. Such advances could broaden this strategy’s applicability for both high-volume industrial manufacturing of TeNPs for photovoltaic and thermoelectric industry or bioremediation efforts in agricultural soils contaminated with toxic oxyanions of metalloids.

## Figures and Tables

**Table 1:** Kinetic parameters for the native substrates of GR from the different enzymes studied.

	GSSG			NADPH		
	*k*_cat_ (s^−1^)	K_M_ (μM)	*k*_cat_/K_M_ (s^−1^ M^−1^)	*k*_cat_ (s^−1^)	K_M_ (μM)	*k*_cat_/K_M_ (s^−1^ M^−1^)
*E. coli* GR	47.3 ± 1.2	82.5 ± 8.2	5.7 × 10^5^	50.9 ± 1	12.7 ± 1	4 × 10^6^
BNF22 GR	377.2 ± 8.8	60.7 ± 4.7	6.2 × 10^6^	141.1 ± 5.6	18.5 ± 2.6	7.6 × 10^6^
MF01 GR	211.9 ± 12.3	266.5 ± 36.7	8 × 10^5^	205 ± 6	63.1 ± 4.9	3.2 × 10^6^
BNF08 GR	115.8 ± 4.2	217.2 ± 21.1	5.3 × 10^5^	139.7 ± 5	41.2 ± 4.2	3.4 × 10^6^

## Data Availability

All data generated and/or analyzed during this study are included in this published article and its Supplementary Materials. All the data are available on request from the corresponding author.

## Supplementary Materials

The following supporting information can be downloaded at: https://www.mdpi.com/article/10.3390/ijms26041549/s1.
