# Supplementary material for: Exploring the Potential of Glutathione Reductase Overexpression to Improve Tellurium Nanoparticle Production in Escherichia coli
Source: Int J Mol Sci. 2025 Feb 12;26(4):1549. doi: 10.3390/ijms26041549 (PMC11855334; doi:10.3390/ijms26041549)
Supplement: Supplementary file 1 [file ijms-26-01549-s001.zip › ijms-3419363-supplementary.pdf]

Supplementary material

Exploring the Potential of Glutathione Reductase Overexpression to Improve Tellurium Nanoparticle Production in *Escherichia coli*

Jefferson O. Romero [1], Víctor Castro-Fernández [2], Estefanía Pérez-Agudelo [3], Fabián Ávila-Salas [3,4,*], Francisco Mura [5] and Felipe Arenas Salinas [1,*]

[1] Departamento de Biología, Facultad de Química y Biología, Universidad de Santiago de Chile, Santiago 9170022, Chile; jefferson.romero@usach.cl
[2] Departamento de Biología, Facultad de Ciencias, Universidad de Chile, Santiago 7800003, Chile; vcasfe@uchile.cl
[3] Centro de Nanotecnología Aplicada, Facultad de Ciencias, Ingeniería y Tecnología, Universidad Mayor, Santiago 8580745, Chile; tefanypa@hotmail.com
[4] Escuela de Agronomía, Facultad de Ciencias, Ingeniería y Tecnología, Universidad Mayor, Santiago 8580745, Chile
[5] Departamento de Ciencias del Ambiente, Facultad de Química y Biología, Universidad de Santiago de Chile, Santiago 9170022, Chile; francisco.mura@usach.cl
* Correspondence: fabian.avila@umayor.cl (F.Á.-S.); felipe.arenass@usach.cl (F.A.S.)

Table S1. Information of strains and plasmids used in this study.

Strain or plasmid	Relevant genotype and characteristic(s)	Reference or source
***E. coli* strains**		
**DH5*α***	F^- φ80*lac*ZΔM15Δ(*lac*ZYA*arg*F)U169 *rec*A1 *end*A1 *hsd*R17(r_K^-, m_K^+) *pho*A *sup*E44 λ^-*thi*-1 *gyr*A96 *rel*A1	Invitrogen™
**MG1655 (DE3)**	Δ*endA* Δ*recA* (DE3)	Addgene (Stranin # 37854)
**C41 (DE3)**	Commercial strain	Lucigen
**C41 (*E. coli* GR)**	Cam^r, C41 strain carrying the *E. coli*-gor plasmid	This work
**C41 (BNF22 GR)**	Amp^r, C41 strain carrying the BNF22 GR plasmid	This work
**C41 (BNF08 GR)**	Amp^r, C41 strain carrying the BNF08 GR plasmid	This work
**C41 (MF01 GR)**	Amp^r, C41 strain carrying the MF01 GR plasmid	This work
**MG1655 (pET101D)**	Amp^r, MG1655 strain carrying pET101/D plasmid	This work
**MG1655 (BNF22 GR)**	Amp^r, MG1655 strain carrying BNF22 GR plasmid	This work
**Plasmids**		
**pET101/D-Topo**	pBR322 ori with T7 RNA polymerase promoter, Amp^r	Invitrogen™
**pET21-b(+)**	pBR322 ori with T7 RNA polymerase promoter, Amp^r	Novagen
***E. coli* GR**	Cam^r, E. Coli GR gene clone in pCA24N plasmid	Nara institute, Japan
**BNF22 GR**	Amp^r, BNF22 GR gene clone in pET101/D-Topo plasmid	Lab stock
**BNF08 GR**	Amp^r, BNF08 GR gene clone in double-digested *Nde*I/ *Hind*III pET21-b (+) plasmid	This work
**MF01 GR**	Amp^r, MF01 GR gene clone in pET101/D-Topo plasmid	Lab stock

Table S2. Distances between relevant atoms involved in the Tellurite Reductase (TR) activity of Glutathione Reductase (GR).

	Average distance 1 Cys47S:HN5FAD	Average distance 2 Cys42S:HN5FAD	Average distance 3 Cys42Cα:Cys47Cα
**BNF22 GR**	4.26 ± 0.09	6.48 ± 0.12	4.75 ± 0.03
***E. coli* GR**	6.19 ± 0.14	8.17 ± 0.13	4.62 ± 0.05
**BNF08 GR**	6.38 ± 0.12	8.36 ± 0.11	4.55 ± 0.04
**MF01 GR**	6.57 ± 0.11	8.61 ± 0.12	4.51 ± 0.03

Distances (Å) were calculated using Maestro from Schrödinger software program.

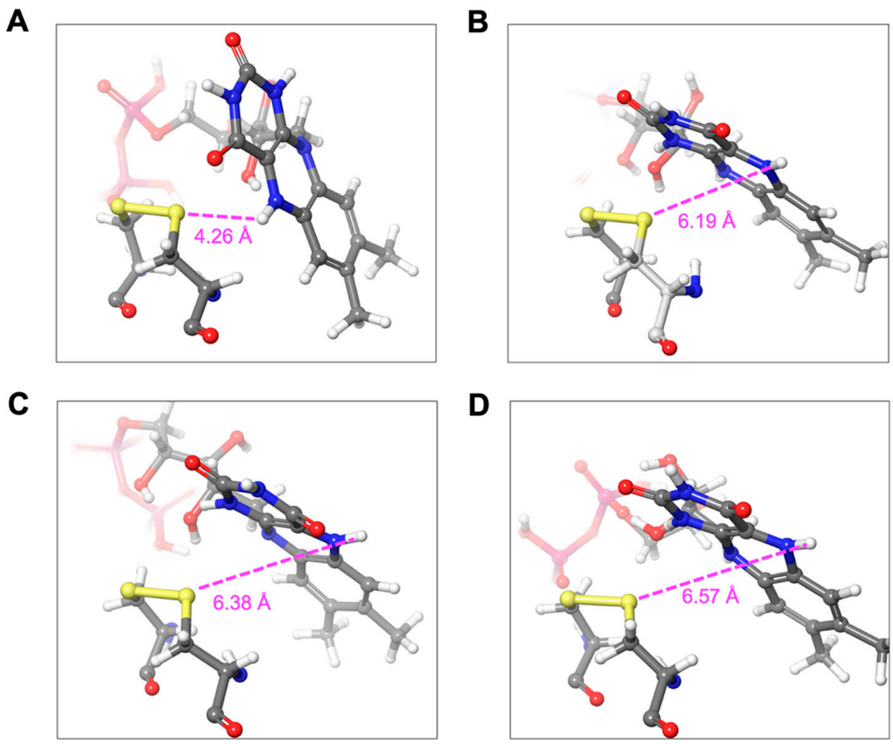

Figure S1. Representative conformations of GR models: (**A**) BNF22 GR, (**B**) *E. coli* GR, (**C**) BNF08 GR and (**D**) MF01 GR illustrating the distance between the hydrogen atom of FADN5 and the sulfur atom of Cys47 during the last 50 ns of the molecular dynamics (MD) simulations. The GR enzymes models were generated with Alphafold 3 web server Beta and relaxed in molecular mechanics (MM) dynamic simulations with the DESMOND program using the force field OPLS_2005. MD simulations were conducted at pH 7.4 in phosphate buffer, GR BNF08 and BNF22 performed at 25 ºC, and *E. coli* GR and MF01 GR at 37 ºC.

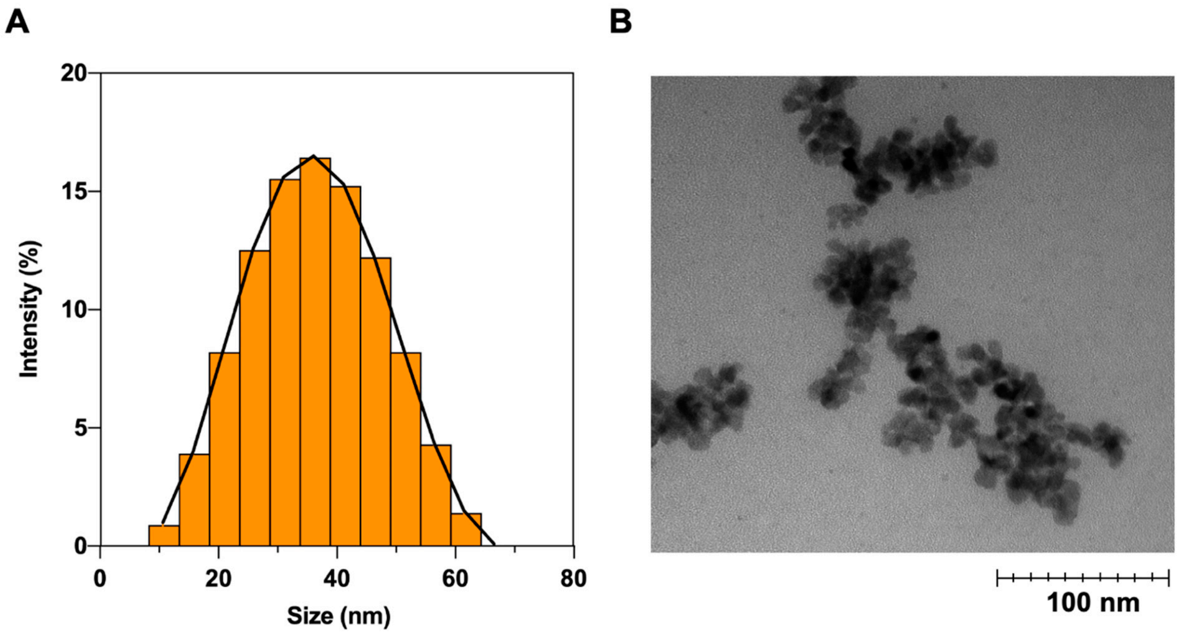

Figure S2. (**A**) Size distribution of purified TeNPs from *E. coli* cultures overexpressing GR form BNF22 analyzed by DLS method. (**B**) Aggregates of purified TeNPs detected by TEM.
